# Access and Awareness of Morbidity Management and Disability Prevention for Lower Limb Lymphatic Filariasis in Post-Mass Drug Administration Districts in Southern India

**DOI:** 10.4269/ajtmh.25-0180

**Published:** 2025-08-05

**Authors:** Anjali Jog, Arpitha Anbu Deborah, Rohan Michael Ramesh, Kumudha Aruldas, Yuvaraj Baskaran, Judd L. Walson, Suma Krishnasastry, Sitara Swarna Rao Ajjampur

**Affiliations:** ^1^ The Wellcome Trust Research Laboratory, Division of Gastrointestinal Sciences, Christian Medical College, Vellore, Tamil Nadu, India;; ^2^ Department of International Health, Bloomberg School of Public Health, Johns Hopkins University, Baltimore, Maryland;; ^3^ WHO Collaborating Centre for Lymphatic Filariasis Morbidity Management and Disability Prevention, Department of Internal Medicine, Govt. T. D. Medical College Hospital, Kerala University of Health Sciences, Alappuzha, Kerala, India

## Abstract

The Global Program to Eliminate Lymphatic Filariasis (LF) aims to eliminate transmission through mass drug administration (MDA) and manage LF disease through morbidity management and disability prevention (MMDP). In this study, surveys on awareness and access to MMDP and quality of life (QoL) among lower limb LF cases in a censused population in two post-MDA districts in Tamil Nadu were conducted. The prevalence of lower limb LF was 0.11% (165/147,871), with 57.6% in reversible stage 1 and 2 categories, 36.4% in stage 3, and 6.1% in stage 4 and above. Among them, 22.4% reported health worker visits, 11.5% were aware of MMDP camps, and 36.4% received MMDP kits. The life activity (mean score: 48.5; SD: 27.3) and mobility (41.2; 24.2) domains exhibited the poorest QoL scores. In post-MDA districts, strengthening the reach and awareness of MMDP programs in communities, especially for early, reversible stages, is pivotal to halting disease progression and improving QoL.

The Global Program to Eliminate Lymphatic Filariasis, which includes mass drug administration (MDA) and morbidity management and disability prevention (MMDP) strategies, was established to eliminate lymphatic filariasis (LF) by 2030.^1^ India currently bears 40% of the global disease burden, spanning 339 LF-endemic districts across 20 states and union territories, impacting ∼481 million people.^2^^,^^3^ In 2004, the Indian government initiated an annual MDA program with diethylcarbamazine citrate (DEC),^4^ which was updated to include DEC–albendazole coadministration in 2006. In 2017, triple-drug therapy, combining ivermectin with DEC and albendazole, was introduced in some districts.^5^^,^^6^ As of 2023, 138 districts in India had cleared transmission assessment surveys (TASs), as defined by the WHO, and are in post-MDA surveillance.^2^^,^^7^ In Tamil Nadu State in southern India, all 20 endemic districts were eligible to stop LF MDA, and the primary focus has been on the MMDP implemented through primary health centers (PHCs) and coordinated by the National Vector-Borne Disease Control Program.^4^ After the successful implementation of LF MDA and the initiation of the MMDP program, the awareness, reach, and effectiveness of MMDP in the community, as well as the quality of life (QoL) of LF cases in post-MDA districts, have not been extensively gauged.^4^ In this study, MMDP awareness, access, and QoL have been assessed among lower limb LF cases in two blocks of two post-MDA districts in Tamil Nadu. This cross-sectional study was conducted in Timiri (Ranipet District, formerly Vellore District) and Jawadhu Hills (Tiruvannamalai District) in Tamil Nadu, southern India. The last round of LF MDA was conducted in Ranipet District in 2013 and in Tiruvannamalai District in 2015, and both districts cleared TAS in 2016 and 2018, respectively.^8^ The primary health needs in these blocks are served by four PHCs and 25 health subcenters (HSCs) in Timiri and three PHCs and 13 HSCs in Jawadhu Hills. As part of a community-based cluster randomized trial for soil-transmitted helminths (Deworm3),^9^ this population has been extensively censused (∼148,000 individuals). The annual census conducted in these blocks from May 2022 to August 2022 was used to record individuals who reported swelling of the lower limbs after obtaining consent from the head of the household. Those reporting lower limb swelling were visited by a medical officer, who, after obtaining written informed consent (or assent for those under 18 years of age), evaluated them using a questionnaire and clinical examination to diagnose and stage lower limb lymphedema (Dreyer seven-stage system) due to LF.^10^ A trained field worker administered a questionnaire on MMDP awareness and QoL to individuals diagnosed with lower limb LF. Sociodemographic characteristics, LF disease status, MMDP awareness and access, and limb hygiene practices of participants were assessed (Table 1). The QoL for each domain was assessed by adapting the WHO Disability Assessment Schedule 2.0 (WHODAS 2.0) and the LF Quality of Life Questionnaire (LFSQQ).^11^ Each domain score (self-care, mobility, life activity, and social life) was analyzed using the WHODAS analysis framework on a scale of 0 to 100, where 100 signifies full disability.^12^ All questionnaires were administered using electronic data capture forms programmed into the SurveyCTO mobile application (Dobility, Cambridge, MA) on encrypted Android smartphones. Student’s *t*-test and one-way analysis of variance were used to compare the QoL scores between domains. The data were analyzed using STATA 18 (STATA Corporation, College Station, TX).

**Table 1:** Sociodemographic characteristics, lymphatic filariasis disease grading, morbidity management and disability prevention awareness and access, and limb hygiene practices among lower limb lymphatic filariasis patients (*N* = 165)

Sociodemographic Characteristics	*n* (%)
Age	
≤60 years	70 (42.4)
>60 years	95 (57.6)
Sex	
Female	101 (61.2)
Male	64 (38.8)
Socioeconomic quintile* (1 = low; 5 = high)	
1	14 (8.5)
2	29 (17.6)
3	40 (24.2)
4	41 (24.8)
5	41 (24.7)
Marital status	
Single/separated/widowed	67 (40.6)
Married	98 (59.4)
Occupation	
Daily wage worker	75 (45.5)
Others	90 (54.6)
Lymphatic filariasis disease grading	
Lower limb affected	
Single leg	149 (90.3)
Both leg	16 (9.7)
Grade of lymphedema (dryer seven-stage system)	
1	11 (6.6)
2	84 (50.9)
3	60 (36.4)
4	7 (4.2)
5	0 (0.0)
6	1 (0.6)
7	2 (1.2)
Acute attacks in last 6 months	108 (65.5)
Fever with swelling	147 (89.1)
Painful lymph nodes with swelling	132 (80.0)
Treatment sought during last acute attack (*n* = 108)	
Doctors and local nurses, unregistered practitioners	96 (88.9)
Self-treatment or home treatment	11 (10.2)
No action	1 (0.9)
Skin morbidity	
Wet lesions/eczema	43 (25.9)
Skin folds	74 (44.6)
Interdigital intertrigo/fungal infection	118 (71.1)
Hair growth	
More than normal limb	10 (6.1)
Less than normal limb	24 (14.5)
Hyperkeratosis	142 (86.1)
Color of affected limb	
Darker than the normal limb	93 (56.0)
Lighter than the normal limb	10 (6.0)
Ulcers	15 (9.1)
Skin knobs/nodules	11 (6.7)
Morbidity management and disability prevention awareness and access	
Aware of any leg hygiene camps	19 (11.5)
Visited by a health worker at the house for the leg swelling	37 (22.4)
Received the MMDP kit^†^	60 (36.4)
MMDP kit last received?	
This year	14 (23.3)
Last year	9 (15.0)
2 years ago	16 (26.7)
3 or more than 3 years ago	21 (35.0)
Currently owns a tub or basin to wash swollen limb	50 (30.3)
Aware of financial assistance offered by government	49 (29.7)
Received financial assistance (*n* = 49)	15 (30.6)
Type of toilet used	
Indian toilet	90 (54.6)
Western toilet	14 (8.5)
No toilet	60 (36.4)
Public toilet	1 (0.6)
Limb hygiene practices	
Wash the affected limb daily	161 (97.6)
Frequency of washing the limb daily (*n* = 161)	
Once	12 (7.5)
Twice	147 (91.3)
Not every day	2 (1.2)
Affected limb washed with	
Water only	78 (48.5)
With soap and water	83 (51.5)
Check for any skin abrasions every day	142 (86.1)
Aware that limb exercises to reduce the severity	15 (9.0)
Exercises everyday (*n* = 15)	8 (53.33)
Aware that limb elevation reduces the severity	67 (40.4)
Elevate affected limb everyday (*n* = 67)	30 (44.8)
Able to wear footwear on the affected limb	150 (90.9)
Type of footwear (*n* = 150)	
Regular footwear	146 (97.3)
Specially made footwear	4 (2.7)
Footwear worn indoors (*n* = 150)	4 (2.7)

MMDP = morbidity management and disability prevention.

* Quintiles, where 1 = low and 5 = high. The wealth index was assessed using quintiles derived from household assets using principal component analysis.

^†^ The MMDP kit contains a tub, a mug, a bucket, a towel, soap, and antifungal cream.

The census identified 747 individuals in 37,691 households with lower limb swelling, of whom 614 (82.2%) were available for re-visit (Figure 1). Among those who consented to clinical examination (559/614), 165 LF-related lower limb cases were identified, resulting in an overall prevalence of 0.11% (165/147,871). Of these cases, 60.8% were women, and the median (interquartile range) age was 62 (53.2–70.2) years. Lower limb LF was more likely in individuals over 60 years of age (adjusted odds ratio [aOR]: 3.8; 95% CI: 2.6–5.5; Supplemental Table 1). Of the 165 patients, 8.5% and 17.6% belonged to the lower socioeconomic quintiles 1 and 2, respectively (Table 1). Lower limb LF was associated with belonging to these lower socioeconomic quintiles (aOR: 2.5; 95% CI: 1.1–5.2 and aOR: 3.6; 95% CI: 1.8–7.5; Supplemental Table 1). More than half (51.5%) of the patients were uneducated or educated only up to primary/middle school. Most identified cases were early-stage LF (stages 1 and 2, 57.6%); 36.4% had moderate lymphedema (stage 3), and 6.1% had advanced lymphedema (stages 4–7). These findings highlight the importance of early MMDP implementation to prevent progression and reduce the burden of disease.^13^ Because a quarter of the lower limb LF cases belonged to the lower socio-economic quintiles, who have poorer access to health services,^14^ and nearly half were daily wage workers (45.5%), community-based sensitization and MMDP activities must emphasize the risk of injury.

**Figure 1.**
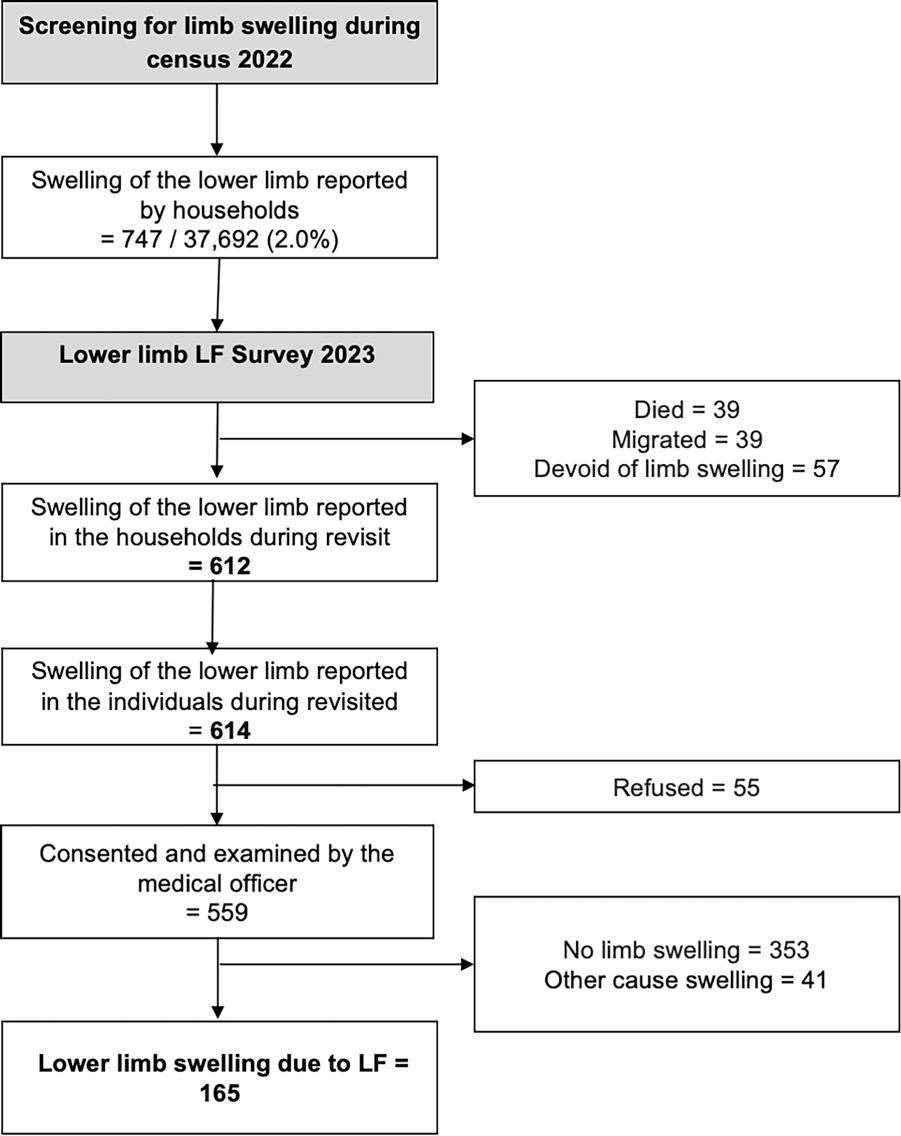
Flow diagram illustrating the number of households screened for any family member with lower limb swelling during a census update of the study area, the number of individuals who consented to have their lower limbs examined by a medical officer, and the number of individuals who were clinically diagnosed with lower limb swelling due to LF.

Skin examination of the affected lower limb revealed that 96.4% (159/165) patients exhibited skin changes, mostly hyperkeratosis (86.1%; Table 1). One or more acute attacks of adenolymphangitis (ADL) were reported by 65.5% of patients in the 6 months preceding the study, with 88.9% seeking care from local doctors, nurses, pharmacists, traditional healers, or quacks. The remainder reported using home-based treatments or did not seek any treatment. This finding highlights the lack of adequate, regular skin care practices and the need to enhance the knowledge and skills of patients and primary care health workers to manage skin conditions in LF and acute ADL.

Among the lower limb LF patients, only 19 (11.5%) were aware of leg hygiene camps organized by the government, 37 (22.4%) reported being visited by a health worker, and 60 (36.4%) reported receiving MMDP kits. Although a high proportion (89.1%) of patients reported practicing limb hygiene twice daily, only 15 (9%) were aware of the role of limb exercises in decreasing disease severity, and only 8 of the 15 reported actively engaging in the exercises (Table 1). Forty percent (67/165) of patients were aware of the benefits of limb elevation, of whom 30 of 67 (44.8%) reported practicing limb elevation daily. Among the patients, 150 (90.9%) reported that they could wear footwear on the affected limb. A few patients (15 [9.1%]) attributed their decision not to wear footwear to challenges in putting them on because of a lack of appropriate sizing. Notably, only four (2.7%) LF patients in stage 3 and above reported owning customized footwear. Awareness of financial assistance offered by the government of Tamil Nadu was noted in 49 (29.7%) cases, with 15 (30.6%) eligible participants (above stage 2) reporting the receipt of financial aid. An earlier study conducted in a post-MDA Tamil Nadu District also revealed that only 48% participants were practicing MMDP regularly, whereas LF stage reversal was limited to 13%, and grade progression was seen in 52%,^4^ highlighting a clear and urgent need for scaling up community MMDP efforts with more contact points and LF-trained healthcare workers. This would facilitate increased compliance with practices of limb hygiene, exercises, and elevation, preventing disease progression and decreasing the burden and direct and indirect costs to already stretched primary healthcare facilities for MMDP.^4^^,^^15^

The mean QoL score assessed was 36.9 (SD: 21.5) among the lower limb LF cases. The reliability of the QoL questionnaire, which was developed by combining the WHODAS and LFSQQ among lower limb LF cases, was validated (Cronbach’s α = 0.97). The life activity domain, which includes household activities and school-related work, exhibited the poorest QoL score of 48.5 (27.3), followed by the mobility domain, which includes standing for 30 minutes, difficulty in moving inside and outside the house, and using public transportation, with a score of 41.2 (24.2). In contrast, the social life domain score and the self-care domain exhibited better QoL scores of 33.9 (21.3) and 26.3 (21.9), respectively. When comparing individual QoL scores across all domains, patients aged 60 years or older, with a clinical grading of LF stage 3 or more, who experienced ADL episodes within the past 6 months had poorer QoL scores. Lymphatic filariasis patients with more severe disease and higher pain scores also had poorer scores across all domains (*P* <0.05; Table 2). This study reveals that even routine tasks at home or work and school were considerably affected, along with mobility inside and outside the house.

**Table 2:** Domain-wise quality of life scores for lower limb lymphatic filariasis cases (*N* = 165)

Domain Score	*n* (%)	Overall		Selfcare		Mobility		Life Activity		Social Life	
		Mean (SD)		Mean (SD)		Mean (SD)		Mean (SD)		Mean (SD)	
		36.9 (21.4)	*P*-Value	26.3 (21.9)	*P*-Value	41.2 (24.2)	*P*-Value	48.5 (27.3)	*P*-Value	33.9 (21.3)	*P*-Value
Age											
≤60 years	70 (42.4)	29.6 (19.7)	0.000	19.2 (17.9)	0.000	31.7 (21.9)	0.000	39.9 (26.9)	0.000	28.3 (21.2)	0.015
>60 years	95 (57.6)	42.2 (21.2)		31.3 (23.3)		47.8 (23.7)		54.5 (25.9)		36.4 (20.8)	
Sex											
Female	101 (61.2)	37.5 (21.7)	0.599	27.0 (22.3)	0.549	42.9 (24.1)	0.188	48.6 (27.1)	0.888	33.5 (21.8)	0.662
Male	64 (38.8)	35.7 (21.2)		25.2 (21.5)		38.3 (24.4)		48.3 (27.7)		32.3 (20.6)	
Clinical grading of lower limb lymphatic filariasis											
1	11 (6.7)	28.8 (18.6)	0.000	16.4 (16.7)	0.001	30.5 (23.5)	0.000	40.1 (29.5)	0.001	28.1 (16.1)	0.019
2	84 (50.9)	31.9 (20.1)		22.7 (18.8)		35.8 (22.3)		42.5 (26.1)		26.7 (20.1)	
3	60 (36.3)	43.9 (20.4)		31.6 (23.9)		48.9 (23.1)		57.4 (24.7)		40.3 (20.2)	
≥4	10 (6.1)	47.8 (27.9)		35.5 (31.6)		51.1 (32.6)		56.6 (35.4)		48.1 (23.2)	
Number of acute episodes of adenolymphangitis											
0	57 (34.5)	28.2 (22.2)	0.000	19.3 (20.4)	0.033	31.8 (25.4)	0.001	36.3 (28.6)	0.001	25.8 (21.6)	0.019
1	28 (16.9)	39.2 (20.3)		28.2 (19.0)		39.2 (23.0)		53.8 (26.9)		35.6 (22.3)	
2	32 (19.4)	42.9 (15.4)		29.6 (18.4)		48.4 (18.3)		57.2 (19.7)		36.4 (17.9)	
>3	48 (29.1)	42.1 (21.9)		31.3 (25.8)		48.6 (23.5)		54.1 (25.8)		37.9 (20.7)	
Duration of limb swelling											
1–10 years	60 (36.3)	32.9 (19.8)	0.288	23.0 (18.7)	0.720	36.2 (23.2)	0.292	43.5 (26.89)	0.246	29.2 (18.5)	0.367
11–20 years	34 (20.6)	40.9 (20.7)		29.0 (21.4)		45.1 (22.4)		55.0 (25.7)		35.6 (21.1)	
21–30 years	26 (15.8)	40.4 (24.8)		27.8 (26.0)		46.0 (26.1)		50.8 (28.7)		36.9 (27.0)	
31–40 years	24 (14.6)	41.0 (20.7)		28.9 (24.6)		45.2 (23.9)		53.9 (24.4)		37.5 (19.8)	
>40 years	21 (12.7)	32.8 (23.1)		26.5 (24.1)		38.6 (27.2)		42.8 (30.6)		30.1 (22.1)	
Disease burden score											
≤33.33	68 (41.2)	24.4 (19.1)	0.000	15.2 (15.6)	0.000	28.0 (22.4)	0.000	34.0 (28.1)	0.000	20.8 (17.3)	0.000
>33.33	97 (58.8)	45.8 (18.5)		34.1 (22.5)		50.4 (21.0)		58.7 (21.5)		41.6 (19.6)	
Pain score											
≤32.5	82 (49.7)	21.8 (15.9)	0.000	12.3 (12.9)	0.000	25.1 (20.4)	0.000	31.1 (24.8)	0.000	20.1 (16.7)	0.000
>32.5	83 (50.3)	51.9 (14.6)		40.2 (20.2)		57.1 (15.8)		65.7 (16.6)		45.9 (17.1)	

Limitations of this study included the fact that home-based care practices were not observed, LF lymphedema was diagnosed and staged, and other uncommon causes of lymphedema were ruled out by clinical examination alone. The strengths of this study included providing estimates of lower limb LF morbidity burden with staging and QoL. The Global Burden of Disease study revealed a 76% decline in disability-adjusted life year/100,000 attributed to the LF MDA program.^16^ Although there are some data on MMDP assessment, an evaluation of footcare practices conducted after an unsupervised period of 1 year or longer in the state of Kerala, India, revealed a reduced frequency of ADL among 72.5% of the study participants, indicating the effectiveness of MMDP programs.^17^ Our findings indicate that attention is needed for LF secondary prevention through hygiene and care practices, tertiary prevention with psychological and socioeconomic support,^18^^,^^19^ and lowering disability through community-based lymphedema management programs.^20^ Morbidity management and disability prevention programs, especially in post-MDA districts, need to create more public awareness, build the capacity of healthcare workers to provide in-person counseling, and engage with and follow up on people living with LF morbidity to encourage proper home-based care. This approach is designed to reduce the frequency and intensity of episodes of ADL, connect individuals to rehabilitation centers and local micro-finance programs, and implement MMDP integrated with LF surveillance. In 2018, the estimated cost of a community-based MMDP program for 2 years in the state of Odisha, India, was $14.68 per patient. However, integrating LF MMDP with limb care for other conditions, such as leprosy and diabetes, may potentially reduce the overall cost of LF MMDP programs.^21^

## Supplemental Materials

10.4269/ajtmh.25-0180 Supplemental Materials

## References

[1] NTD Modelling Consortium Lymphatic Filariasis Group, 2019. The roadmap towards elimination of lymphatic filariasis by 2030: Insights from quantitative and mathematical modelling. Gates Open Res 3: 1538. 31728440 10.12688/gatesopenres.13065.1 PMC6833911

[2] National Centre for Vector Borne Diseases Control, Ministry of Health and Family Welfare, Government of India, 2023. Newsletter on Lymphatic Filariasis. Available at: https://ncvbdc.mohfw.gov.in/Doc/VL-LF-Newsletter-December-2023.pdf. Accessed March 17, 2025.

[3] Pati S, 2023. Eliminating lymphatic filariasis: India’s bold plan to finish 3 years ahead of global schedule. Indian J Public Health 67: 345–346. 37929372 10.4103/ijph.ijph_1030_23

[4] Mathiarasan L Das LK Krishnakumari A, 2021. Assessment of the impact of morbidity management and disability prevention for lymphatic filariasis on the disease burden in Villupuram District of Tamil Nadu, India. *Indian J Community Med Off Med* 46: 657–661. 10.4103/ijcm.IJCM_12_21 PMC8729271 35068729

[5] Kumar SP, 2020. Lymphatic filariasis in India: A journey towards elimination. J Commun Dis 52: 17–21.

[6] Tripathi B Roy N Dhingra N, 2022. Introduction of triple-drug therapy for accelerating lymphatic filariasis elimination in India: Lessons learned. Am J Trop Med Hyg 106: 29–38. 35292580 10.4269/ajtmh.21-0964 PMC9154644

[7] Sharma S Smith ME Bilal S Michael E, 2023. Evaluating elimination thresholds and stopping criteria for interventions against the vector-borne macroparasitic disease, lymphatic filariasis, using mathematical modelling. Commun Biol 6: 225. 36849730 10.1038/s42003-022-04391-9 PMC9971242

[8] DeWorm3 Trials Team, 2020. Baseline patterns of infection in regions of Benin, Malawi and India seeking to interrupt transmission of soil transmitted helminths (STH) in the DeWorm3 trial. PLoS Negl Trop Dis 14: e0008771. 33137100 10.1371/journal.pntd.0008771 PMC7673551

[9] Ásbjörnsdóttir KH, ; DeWorm3 Trials Team, 2018. Assessing the feasibility of interrupting the transmission of soil-transmitted helminths through mass drug administration: The DeWorm3 cluster randomized trial protocol. PLoS Negl Trop Dis 12: e0006166. 29346377 10.1371/journal.pntd.0006166 PMC5773085

[10] National Centre for Vector Borne Diseases Control, Ministry of Health and Family Welfare, Government of India Lymphatic Filariasis. Available at: https://ncvbdc.mohfw.gov.in/WriteReadData/l892s/43461824631532409675.pdf. Accessed March 17, 2025.

[11] Thomas C Narahari SR Bose KS Vivekananda K Nwe S West DP Kwasny M Kundu RV, 2014. Comparison of three quality of life instruments in lymphatic filariasis: DLQI, WHODAS 2.0, and LFSQQ. PLoS Negl Trop Dis 8: e2716. 24587467 10.1371/journal.pntd.0002716 PMC3930502

[12] Üstün TB, 2010. Measuring Health and Disability: Manual for WHO Disability Assessment Schedule (WHODAS 2.0). Available at: https://www.who.int/publications/i/item/measuring-health-and-disability-manual-for-who-disability-assessment-schedule-(-whodas-2.0). Accessed September 8, 2025.

[13] World Health Organization, 2021. *Lymphatic Filariasis: Managing the Disease and Its Complications*. Available at: https://www.who.int/publications/i/item/9789240017061. Accessed March 17, 2025.

[14] Rao PS Richard J, 1984. Socioeconomic and demographic correlates of medical care and health practices. J Biosoc Sci 16: 343–355. 6470017 10.1017/s0021932000015169

[15] Gyapong JO Gyapong M Evans DB Aikins MK Adjei S, 1996. The economic burden of lymphatic filariasis in northern Ghana. Ann Trop Med Parasitol 90: 39–48. 8729626 10.1080/00034983.1996.11813024

[16] Dutta O Prasanth A Kumari A Akanksha K Deeba F Salam N, 2023. Burden of dengue, leishmaniasis and lymphatic filariasis in India and its states from 1990–2019: Analysis from the Global Burden of Disease study (GBD 2019). PLoS One 18: e0292723. 37851660 10.1371/journal.pone.0292723 PMC10584127

[17] Suma TK Shenoy RK Kumaraswami V, 2002. Efficacy and sustainability of a footcare programme in preventing acute attacks of adenolymphangitis in Brugian filariasis. Trop Med Int Health 7: 763–766. 12225507 10.1046/j.1365-3156.2002.00914.x

[18] World Health Organization, 2003. *Learner’s Guide: Training Module on Community Home-Based Prevention of Disability Due to Lymphatic Filariasis*. Available at: https://iris.who.int/bitstream/handle/10665/67873/WHO_CDS_CPE_CEE_2003.35_Part1.pdf?sequence=1. Accessed March 17, 2025.

[19] Kumari AK Jayaraman Y Das LK, 2012. Issues in delivering morbidity management for lymphatic filariasis elimination: A study in Pondicherry, South India. ScientificWorldJournal 2012: 372618. 22654597 10.1100/2012/372618 PMC3361224

[20] Budge PJ Little KM Mues KE Kennedy ED Prakash A Rout J Fox LM, 2013. Impact of community-based lymphedema management on perceived disability among patients with lymphatic filariasis in Orissa State, India. *PLoS Negl Trop Dis* 7: e2100. 23516648 10.1371/journal.pntd.0002100 PMC3597476

[21] Sawers L Stillwaggon E, 2020. Economic costs and benefits of community-based lymphedema-management programs for lymphatic filariasis in India. Am J Trop Med Hyg 103: 295–302. 32653050 10.4269/ajtmh.19-0898 PMC7356420

