# Supplementary material for: Access and Awareness of Morbidity Management and Disability Prevention for Lower Limb Lymphatic Filariasis in Post-Mass Drug Administration Districts in Southern India
Source: Am J Trop Med Hyg. 2025 Aug 5;113(4):839–43. doi: 10.4269/ajtmh.25-0180 (PMC12493119; doi:10.4269/ajtmh.25-0180)
Supplement: Supplemental Materials [file tpmd250180.SD1.pdf]

Supplemental Table 1

Multilevel Logistic Regression Analysis for Factors Associated with lower limb lymphatic filariasis cases (*N* = 165)

Characteristics	No Morbidity	LF Morbidity	Adjusted OR (95% CI)	*P*-Value
**Education**				
High school/College	25,303 (23.5)	10 (6.1)	Ref	Ref
Secondary	20,295 (18.7)	17 (10.4)	1.7 (0.7 - 3.9)	0.176
Primary /Middle	34,415 (31.9)	85 (52.2)	3.6 (1.8 - 7.5)	**0.000**
No education	27,601 (25.7)	51 (31.3)	2.5 (1.1 – 5.2)	**0.025**
**Age**				
<=60 years	91,786 (62.1)	68 (41.2)	Ref	Ref
>60 years	55,920 (37.9)	97 (58.8)	3.8 (2.6 – 5.5)	**0.000**
**Sex**				
Male	73,694 (49.9)	64 (38.8)	Ref	Ref
Female	73,993 (50.1)	101 (61.2)	1.2 (0.9 – 1.8)	0.201
**Marital Status**				
Unmarried	25,614 (39.2)	7 (4.2)	Ref	Ref
Married	76,686 (66.1)	102 (61.8)	1.1 (0.5 – 2.5)	0.832
Separated/Divorced/Widow	13,704 (11.8)	56 (33.9)	1.5 (0.4 – 2.2)	0.402
**Caste**				
Schedule tribe/Schedule caste	76,381 (51.9)	149 (90.3)	Ref	Ref
Backward/Most backward	70,707 (48.1)	16 (9.7)	5.4 (2.9 – 9.8)	**0.000**

OR – odds ratio, aOR – adjusted odds ratio, 95% CI – 95% confidence interval
A multilevel logistic regression model was developed to examine factors associated with LF disease. This model incorporated sociodemographic variables, including education, marital status, house type, quintiles, caste, age, and sex. The final model was selected based on the Akaike Information Criterion (AIC) and Schwarz's Bayesian Information Criterion (BIC), with statistical significance set at $P < 0.05$. Adjusted odds ratios (aORs) with 95% confidence intervals were calculated to assess the associations between variables.
